# Anthropometric, biochemical and clinical assessment of malnutrition in Malaysian patients with advanced cirrhosis

**DOI:** 10.1186/1475-2891-9-27

**Published:** 2010-06-24

**Authors:** Mei-Ling S Tai, Khean-Lee Goh, Siti Hawa Mohd-Taib, Sanjay Rampal, Sanjiv Mahadeva

**Affiliations:** 1 Department of Medicine, Faculty of Medicine, University of Malaya, Kuala Lumpur, 50603, Malaysia; 2 Department of Dietitics, University Malaya Medical Centre, Jalan University, Kuala Lumpur 59100, Malaysia; 3 Department of Social and Preventive Medicine, Faculty of Medicine, University of Malaya, Kuala Lumpur 50603, Malaysia

## Abstract

**Background:**

There is limited data on the nutritional status of Asian patients with various aetiologies of cirrhosis. This study aimed to determine the prevalence of malnutrition and to compare nutritional differences between various aetiologies.

**Methodology:**

A cross-sectional study of adult patients with decompensated cirrhosis was conducted. Nutritional status was assessed using standard anthropometry, serum visceral proteins and subjective global assessment (SGA).

**Results:**

Thirty six patients (mean age 59.8 ± 12.8 years; 66.7% males; 41.6% viral hepatitis; Child-Pugh C 55.6%) with decompensated cirrhosis were recruited. Malnutrition was prevalent in 18 (50%) patients and the mean caloric intake was low at 15.2 kcal/kg/day. SGA grade C, as compared to SGA grade B, demonstrated significantly lower anthropometric values in males (BMI 18.1 ± 1.6 vs 26.3 ± 3.5 kg/m2, p < 0.0001; MAMC 19.4 ± 1.5 vs 24.5 ± 3.6 cm, p = 0.002) and females (BMI 19.4 ± 2.7 vs 28.9 ± 4.3, p = 0.001; MAMC 18.0 ± 0.9 vs 28.1 ± 3.6, p < 0.0001), but not with visceral proteins. The SGA demonstrated a trend towards more malnutrition in Child-Pugh C compared to Child-Pugh B liver cirrhosis (40% grade C vs 25% grade C, p = 0.48). Alcoholic cirrhosis had a higher proportion of SGA grade C (41.7%) compared to viral (26.7%) and cryptogenic (28.6%) cirrhosis, but this was not statistically significant.

**Conclusion:**

Significant malnutrition in Malaysian patients with advanced cirrhosis is common. Alcoholic cirrhosis may have more malnutrition compared to other aetiologies of cirrhosis.

## Introduction

Cirrhosis of the liver is a devastating condition, commonly the result of decades of chronic inflammation from toxin (eg alcohol), viral infection (eg Hepatitis B) or immune mediated disease (eg autoimmune disease). As a result of the complex pathophysiological processes associated with cirrhosis, it results in significant morbidity such as gastrointestinal bleeding from portal hypertension, and eventual mortality in many patients [1]. The prognosis of patients with advanced cirrhosis is grim, with a 5-year survival rate of <10%. Patients with decompensated liver cirrhosis form the majority of cases that are admitted into gastroenterology units world-wide and represent a significant burden on health-care resources [2].

In addition to the associated morbidity highlighted above, protein-energy malnutrition (PEM) has often been observed in patients with liver cirrhosis [3,4]. Previous studies in Western patients have documented malnutrition rates from 20% in compensated liver cirrhosis up to 60% in decompensated liver cirrhosis [5]. Causes for malnutrition in liver cirrhosis are known to include a reduction in oral intake (for various causes), increased protein catabolism and insufficient synthesis, and malabsorption/maldigestion associated with portal hypertension [3,5,6]. Although a consequence of the disease, malnutrition alone can lead to further morbidity in patients with liver cirrhosis. Increased rates of septic complications, poorer quality of life, and a reduced life span have all been observed in cirrhotics with poorer nutrition status compared to those without [7,8].

In Asia, the high prevalence of chronic Hepatitis B infection, has resulted in large numbers of people developing liver cirrhosis with its' associated complications [9]. Most of the data on malnutrition in patients with cirrhosis have been derived from Western patients in whom chronic alcohol ingestion has been the commonest aetiology. Alcoholic patients are known to develop malnutrition for other reasons apart from liver damage per se [10]. It is uncertain, therefore, if Asian patients with cirrhosis have the same degree of malnutrition and its' resultant morbidity as patients with cirrhosis from other parts of the world.

The aims of this study were: a) to determine the prevalence of malnutrition in Malaysian patients with cirrhosis using standard nutritional assessment tools and b) to compare nutritional differences between various aetiologies.

## Methods

### Patients

Local institutional ethics committee approval was sought before commencement of the study. A cross-sectional study of Asian patients admitted for decompensation of cirrhosis to this tertiary institution, between August 2006 and March 2007, was undertaken. The inclusion criteria were adults aged 18 years and above, admitted for the reason of decompensation of cirrhosis. Patients with hepatocellular carcinoma and severe, i.e. Grade 3 or 4, hepatic encephalopathy were excluded. Eligible patients were given an information sheet in both English and Malay language detailing the objectives and nature of the study. Informed consent was obtained in all patients prior to participation.

### Clinical criteria

Cirrhosis was diagnosed based on a combination of clinical features, blood profile and radiological imaging. Clinical features were those of portal hypertension, i.e. ascites and/or gastrointestinal varices. Blood profile included evidence of thrombocytopenia and/or coagulopathy. Radiological features, either with trans-abdominal ultrasound or computerized tomography, had to demonstrate a small shrunken liver with or without splenomegaly and intra-abdominal varices. Severity of liver disease was calculated according to the Child-Pugh score with grades A (mild) to C (severe) indicating degree of hepatic reserve and function [11].

### Nutritional Assessment

Nutritional assessment was based on the following: anthropometry, visceral proteins, lean body mass and subjective global assessment (SGA). All measurements were taken by the same single investigator, to avoid any inter-observer variation.

#### Anthropometry

All patients in the study had a baseline body mass index (BMI), i.e. weight (kg)/height (meter) ² performed. Although a crude measure of nutritional status, BMI was used as a baseline comparison between cirrhotic patients and the local healthy population [12]. Further anthropometric measurements included the following: midarm circumference (MAC), triceps skinfold thickness (TST), midarm muscle circumference (MAMC) and handgrip strength. MAC was measured to the nearest centimeter with a measuring tape at the right arm. TST, an established measure of fat stores, was measured to the nearest millimeter at the right arm using Harpenden skinfold caliper (Baty Ltd, British Indicators) in a standard manner [13]. Three measurements were taken for both TST and MAC, with average values calculated and recorded. Mid-arm muscle circumference (MAMC), an established measure of muscle protein mass, was calculated from MAC and TST using a standard formula: MAMC = MAC - (3.1415*TSF) [13].

Handgrip strength, a simple and effective tool to measure nutritional status, was measured with a hydraulic hand dynamometer (JAMAR) in kilogram force (Kg/F) [14]. Three measurements were made on each arm and an average taken from all measurements. A combination of handgrip strength <30 kg/F and MAMC <23 cm had previously been shown to have a 94% sensitivity and 97% negative predictive value in identifying malnourished patients [15].

#### Visceral proteins

Serum albumin concentration is the most frequently used laboratory measure of nutritional status. Although non-specific, it has been used to assess change in nutritional status and stratifying risk of malnutrition [16]. A reduction in serum albumin in the absence of other causes has been shown to represent liver damage and hence forms part of the normal items for a classic liver function test. Serum transferrin has a half-life of 9 days, and can be used as a marker for malnutrition. Good correlation between transferrin level with the Child-Pugh score has been demonstrated before [17] and a reduced level of serum transferrin is additionally indicative of decreased caloric intake.

#### Subjective global assessment

Subjective global assessment (SGA) is a simple evaluation tool that allows physicians to incorporate clinical findings and subjective patient history into a nutritional assessment [17]. Based on history taking and physical examination, nutritional ratings of patients are obtained as follows: well-nourished-A, moderately malnourished-B and severely malnourished-C. The SGA has been shown to be a valid and useful clinical nutritional assessment tool for patients of various medical conditions [18].

#### Malnutrition

Malnutrition was defined as <5th percentile MAMC for purposes of standardization with the literature and for accurate comparisons with other cirrhotic populations [15]. However, it is recognized that other markers of malnutrition such hand grip strength [15] and SGA [18] have been used, albeit in fewer studies.

#### Dietary intake and assessment

Assessment of individual patient's oral intake during hospitalization was determined by the dietary recall method done every three days for two weeks and an average intake was calculated and recorded. The objective was to determine the adequacy of caloric intake per patient with minimum reporting bias. Calculation of calories of food and drinks intake (composition of the diet) was based on local reference data [19].

### Statistical Analysis

All data was entered into Statistical Packages for the Social Sciences (SPSS) version 13.0 (Chicago, Illinois, USA) software for analysis. Continuous variables were expressed as means with standard deviation and analysed with student's t-test or Mann-Whitney where appropriate whilst categorical data were analysed using the χ2 test. For the comparison of nutritional status in cirrhotic patients of various etiologies, SGA was also utilized as this has been shown to reliably identify malnutrition-related muscle dysfunction [20]. Statistical significance was assumed at a p value of <0.05.

## Results

A total of 36 patients with decompensated liver cirrhosis were recruited during the study period. The basic demography and clinical features are highlighted in Table 1. The mean age of the patients was 59.8 ± 12.8 years and the most common reason for admission was tense ascites requiring paracentesis. Viral hepatitis (n = 15, 41.6%) and alcoholic liver disease (n = 12, 33.3%) were the most common aetiology of cirrhosis. 7/12 patients with alcoholic liver disease had an active alcohol intake at the time of the study. All patients had advanced liver disease with 16 (44.4%) cases of Child-Pugh B and 20 (55.6%) cases of Child-Pugh C cirrhosis.

**Table 1:** Patient profile

	Patients with cirrhosis n = 36	
**Age**	59.8 ± 12.8 years	

**Gender (Male: Female)**	1.8: 1	

**Ethnicity**	Malay n = 11 (30.6%) Chinese n-14 (38.9%) Indian n = 11 (30.6%)	

**Child-Pugh grade**	Child Pugh B n = 16 (44.4%) Child Pugh C n = 20 (55.6%)	

**Aetiology/Child-Pugh grade C**	Alcohol n = 12 (33.3%)	8/12
	Viral hepatitis n = 15 (41.6%)	10/15
	Autoimmune n = 2 (5.6%)	1/2
	Cryptogenic n = 7 (19.4%)	1/7

**Reason for admission**	Tense ascites with need for paracentesis n = 15 (41.7%) Infection n = 7 (19.4%) Diarrhoea n = 3 (8.3%) Abdominal pain n = 2 (5.6%) Upper gastrointestinal bleed n = 2 (5.6%) Diabetic ketoacidosis n = 2 (5.6%) Others n = 5 (13.9%)	

Mean CRP (mg/litre)	4.54 ± 4.87	

BMI (kg/m²)	24.3 ± 5	

Caloric Intake (Kcal/kg/day)	15.2 kcal/kg/day	

### Nutritional assessment

Malnutrition, i.e. MAMC < 5th percentile, was prevalent in 18/36 (50%) patients and the mean caloric intake of all cirrhotic patients was low at 15.2 kcal/kg/day. Biochemically, the mean serum albumin (20.6 ± 6.0 g/l) and the mean serum transferrin (1.6 ± 0.7 g/l) were lower than normal values. 24 (66.7%) patients had SGA Grade B nutritional status and 12 (33.3%) were SGA Grade C, i.e. all patients had some level of malnutrition based on the SGA scale.

Table 2 illustrates the nutritional parameters of the subjects, according to SGA grades. As expected, in both male and female patients with cirrhosis, mean values of anthropometric measurements such as BMI, MAMC and TST demonstrated significant differences in values between cirrhotic patients in SGA grades B and C, with a higher SGA grade correlating well with lower anthropometric values. However, this difference was not observed with visceral proteins such as serum albumin or transferrin (Table 2).

**Table 2:** Nutritional parameters in patients with cirrhosis according to gender and Subjective Global Assessment grades

	**SGA B	**SGA C	p value #	Normal values
	
	Male patients with cirrhosis (n = 23)			
BMI (kg/m2)	26.3 ± 3.5	18.1 ± 1.6	<0.0001	23.8

*MAMC (cm) (% < 5^th ^percentile)	24.5 ± 3.6 (21.7)	19.4 ± 1.5 (39.1)	0.002	23.6

*TST (mm) (% < 5^th ^percentile)	10.1 ± 3.1 0	5.9 ± 1.4 (4.3)	0.003	5

Handgrip strength (kgF)	20.8 ± 7.9	16.1 ± 6.2	0.18	34.1

Albumin (g/l)	20.9 ± 7.4	23.0 ± 5.0	0.51	35-50

Transferin (g/l)	1.7 ± 0.7	1.5 ± 0.8	0.68	1.8-2.7

	**Female patients with cirrhosis (n = 13.)**			

BMI (kg/m2)	28.9 ± 4.3	19.4 ± 2.7	0.001	24.6

MAMC (cm) (% < 5^th ^percentile)	28.1 ± 3.6 (0)	18.0 ± 0.9 (30.8)	<0.0001	18.5

TST (mm) (% < 5^th ^percentile)	10.9 ± 4.2 (33.3)	8.6 ± 1.1 (33.3)	0.24	12

Handgrip strength (kgF)	13.4 ± 5.2	9.1 ± 2.2	0.06	34.1

Albumin (g/l)	20.1 ± 4.4	17.2 ± 2.9	0.22	35-50

Transferin (g/l)	1.9 ± 0.5	1.2 ± 0.4	0.03	1.8-2.7

*BMI = body mass index; TST = triceps skinfold thickness; MAMC = mid-arm muscle circumference

** Subjective Global Assessment

# Student's T-test

Differences in nutritional status in Child-Pugh B and C liver cirrhosis was assessed with the SGA (Table 3). There was a higher proportion of patients with SGA grade C in Child-Pugh C cirrhotic compared to Child-Pugh B cirrhotic patients, although this was not statistically significant ( 40% vs 25%, p = 0.48). However, serum albumin (17.9 ± 4.4 vs 24.1 ± 6.0 g/L, p = 0.001) and transferrin (1.3 ± 0.6 vs 2.0 ± 0.5 g/L, p < 0.0001) levels were demonstrated to be significantly lower in patients with Child-Pugh C liver cirrhosis compared to those with Child-Pugh B disease. Caloric intake was further observed to be significantly less in patients with Child-Pugh C disease compared to patients with Child-Pugh B disease (13.3 ± 4.9 vs 17.6 ± 5.7 Kcal/kg/day, p = 0.018).

**Table 3:** Subjective Global Assessment in varying severity and aetiologies of cirrhosis

	SGA grade B n (%)	SGA grade C n (%)	p value #
**Severity of cirrhosis**			
Child-Pugh B (n = 16)	12 (75)	4 (25)	0.48
Child-Pugh C (n = 20)	12 (60)	8 (40)	

**Aetiology of cirrhosis**			
Viral Hepatitis (n = 15)	11 (73.3)	4 (26.7)	0.93
Alcoholic (n = 12)	7 (58.3)	5 (41.7)	0.57
Cryptogenic (n = 7)	5 (71.4)	2 (28.6)	0.81
Autoimmune (n = 2)	1 (50)	1 (50)	0.58

# Chi-square test

### Aetiology of liver disease and nutritional parameters

The incidence of malnutrition, defined as % < 5^th ^percentile MAMC, in the different aetiologies of cirrhosis were as follows: Alcoholic liver disease n = 9/12 (75%), ViralHepatitis (Hepatitis B & C combined) n = 5/15 (33.3%), Cryptogenic n = 2/7 (28.6%) and Autoimmune n = 1/2 (50%). Differences in nutritional status between the various aetiologies of cirrhosis were examined with the SGA (Table 3). Excluding the extremely small number of autoimmune cirrhotic patients, there was a non-statistically significant increase in the proportion of SGA grade C cases in patients with an alcoholic aetiology (41.7%), compared to those with a viral (26.7%) and cryptogenic (28.6%) aetiology for cirrhosis.

## Discussion

This study of nutritional assessment in Malaysian patients with advanced cirrhosis has several limitations. The sample size was small, resulting in some limitations with the relevance of the results from the study. Furthermore, the study was conducted on a selected group of patients with cirrhosis, namely those with advanced end-stage disease who had been admitted to hospital for decompensation. Additionally, a significant proportion of patients with ascites did not have dry weight measurements done, which could have influenced BMI and calorie calculation results. Nevertheless, this study provides useful nutritional data which is currently lacking among Asian patients with advanced cirrhosis.

This study demonstrated that the prevalence of malnutrition, defined by MAMC < 5^th ^percentile, was 50% in Malaysian patients with advanced cirrhosis. The patients with cirrhosis exhibited a range of nutritional abnormalities, with protein-energy malnutrition of 50% (MAMC < 5^th ^percentile) and fat store depletions of 30% (TST <5^th ^percentile). BMI measurements in less malnourished cirrhotic patients were not different from the general population, mainly due to the fact that ascites and peripheral oedema contributed significantly to body weight in cirrhotic patients, and true lean body mass was not taken into account [21]. The poor caloric intake of 15.2 kcal/kg/day is lower than the recommended level (24 - 40 kcal/kg/day [22]), and may have been one of the causes of this malnutrition, although other factors are well recognized [3,5].

The level of malnutrition identified in this study appears to be comparable to published data from Italy (34% of cirrhotics with MAMC < 5^th ^percentile) [7], a hospital-based study of 315 patients from France (58.7% of Child-Pugh C cirrhotic patients with MAMC < 5^th ^percentile) [23] and a previous study from Thailand (38% of cirrhotics with TSF <10^th ^percentile) [24]. This data suggests that nutritional deficiencies in cirrhosis are likely to be uniform worldwide, regardless of the ethnic distribution or socioeconomic status (believed to be higher in Western patients compared to Asians) of the population involved.

This study further supported the utility of the SGA in Asian patients with cirrhosis. Although anthropometric tools such as the MAMC and hand grip strength are known to be better predictors of malnutrition in adult patients with cirrhosis [15], these tools are not necessarily practical for everyday use. The SGA, compared to standard anthropometry, is much more applicable in clinical practice and has previously been demonstrated to be highly predictive of malnutrition in advanced cirrhosis [24]. We demonstrated in this study that SGA grade C patients with cirrhosis had significantly lower anthropometric measurements compared to SGA grade B cases, indicating that the SGA was able to differentiate nutritional status fairly well.

In terms of clinical severity, we were able to demonstrate a trend towards a higher proportion of SGA grade C in patients with Child-Pugh C cirrhosis compared to Child-Pugh B disease. The lack of statistical significance in this observation was probably a result of the small sample size of our study population, i.e. a Type II statistical error. Furthermore, the caloric intake in patients with more advanced cirrhosis was significantly lower with a likelihood of more malnutrition in this group. In this study, we demonstrated that serum visceral protein levels did not differ significantly between SGA Grade B and C, but varied markedly between Child-Pugh B and C liver disease. This indicated that visceral proteins were not influenced by nutritional status but more by the severity of hepatic dysfunction [25].

Differences in malnutrition between various aetiologies of cirrhosis were explored in this study. The frequency of malnutrition in alcohol-related cirrhosis was higher than other aetiologies and the SGA demonstrated a trend towards more severe malnutrition in adults with alcoholic cirrhosis compared to other types of cirrhosis. The latter was not statistically significant, probably as result of the small number of patients in this study. One of the possible explanations for this finding was that 7/12 alcoholic patients were still actively consuming alcohol at the time of the study, leading to more severe nutritional deficiencies in these patients as previously reported [10]. Our findings are in agreement with studies that have been conducted in larger populations. In a study of 1402 patients with cirrhosis in Italy, there was a higher incidence of malnutrition in alcoholic cirrhosis patients compared to other aetiologies of liver cirrhosis [26]. In a Thai study of 60 patients with cirrhosis, the degree of malnutrition was higher in patients with alcoholic cirrhosis and these patients had more complications of cirrhosis compared to other aetiologies [24].

## Conclusions

In summary, malnutrition in Malaysian patients with various aetiologies of cirrhosis is common, together with an inadequate caloric intake. Clinical assessment with the SGA demonstrated a trend towards more malnutrition with increasing clinical severity and in alcohol related liver disease, although this was not statistically significant. Serum visceral proteins were not found to be an appropriate tool for nutritional assessment in adults with decompensated cirrhosis. A study with a larger sample is required to substantiate these findings.

## Competing interests

The authors declare that they have no competing interests.

## Authors' contributions

MLST designed the study, performed data collection, data analysis and drafted the manuscript. KLG provided administrative support. SHMT provided technical support. SR assisted in data analysis and interpretation. SM assisted in data interpretation and critical revision of the manuscript. All authors reviewed and approved final version of the manuscript.
